# A Multiallelic Molecular Beacon-Based Real-Time RT-PCR Assay for the Detection of SARS-CoV-2

**DOI:** 10.3390/life11111146

**Published:** 2021-10-27

**Authors:** Andreas C. Chrysostomou, Johana Hezka Rodosthenous, Cicek Topcu, Christina Papa, Antonia Aristokleous, Georgia Stathi, Christina Christodoulou, Christina Eleftheriou, Dora C. Stylianou, Leondios G. Kostrikis

**Affiliations:** 1 Department of Biological Sciences, University of Cyprus, Aglantzia, Nicosia 2109, Cyprus; chrysostomou.c.andreas@ucy.ac.cy (A.C.C.); rodosthenous.johana@ucy.ac.cy (J.H.R.); topcu.m.cicek@ucy.ac.cy (C.T.); papa.christina@ucy.ac.cy (C.P.); aristokleous.antonia@ucy.ac.cy (A.A.); stathi.georgia@ucy.ac.cy (G.S.); cc2018@cam.ac.uk (C.C.); stylianou.c.dora@ucy.ac.cy (D.C.S.); 2 Department of Health and Safety, University of Cyprus, Aglantzia, Nicosia 2109, Cyprus; celeft03@ucy.ac.cy

**Keywords:** COVID-19, SARS-CoV-2, molecular beacons

## Abstract

Emerging infectious viruses have led to global advances in the development of specific and sensitive detection techniques. Viruses have an inherent potential to easily mutate, presenting major hurdles for diagnostics and requiring methods capable of detecting genetically diverse viral strains. One such infectious agent is severe acute respiratory syndrome coronavirus 2 (SARS-CoV-2), which emerged in December 2019 and has resulted in the global coronavirus disease 2019 (COVID-19) pandemic. This study presents a real-time reverse transcription PCR (RT-PCR) detection assay for SARS-CoV-2, taking into account its intrinsic polymorphic nature that arises due to genetic drift and recombination, as well as the possibility of continuous and multiple introductions of genetically nonidentical strains into the human population. This advance was achieved by using mismatch-tolerant molecular beacons designed to specifically detect the SARS-CoV-2 S, E, M, and N genes. These were applied to create a simple and reproducible real-time RT-PCR assay, which was validated using external quality control panels (QCMD: CVOP20, WHO: SARS-CoV-2-EQAP-01) and clinical samples. This assay was designed for high target detection accuracy and specificity and can also be readily adapted for the detection of other emerging and rapidly mutating pathogens.

## 1. Introduction

Infectious diseases caused by pathogenic agents are among the most prominent leading causes of death worldwide. In the last few decades alone, humanity has experienced the emergence and/or re-emergence of pathogens, which has led to epidemics and pandemics, such as those caused by human immunodeficiency virus (HIV), influenza, severe acute respiratory syndrome (SARS), and Ebola. Evidently, viral pathogens are more often than not the causative agents of emerging infections [1,2]. Currently, the world is facing yet another pandemic, which was initially identified as an outbreak of pneumonia cases of an unknown cause in December 2019 in Wuhan city, the capital of Hubei Province in China. This respiratory disease was named coronavirus disease 2019 (COVID-19), after its causative agent, which was revealed to be a novel coronavirus named SARS coronavirus 2 (SARS-CoV-2) [3]. SARS-CoV-2 is the third zoonotic human coronavirus that can cause severe disease, with the other two being SARS-CoV and Middle East respiratory syndrome coronavirus (MERS-CoV) [4,5,6,7].

As a member of the *Coronaviridae* family, SARS-CoV-2 has a positive-sense, single-stranded ribonucleic acid (RNA) genome of ∼ 30,000 base pairs (bp), the largest genome of all RNA viruses that infect humans. Through sequencing and phylogenetic studies, SARS-CoV-2 was classified in the genus *Betacoronavirus* along with SARS-CoV and MERS-CoV [8]. It has been discovered that the SARS-CoV-2 genome has 14 open reading frames (ORFs), with the first being ORF1ab, which encodes 15–16 nonstructural proteins (nsps) and mainly comprises the replicase/transcriptase complex that is important for viral replication [9]. Following ORF1ab, the four main structural proteins are encoded: spike (S), envelope (E), membrane (M), and nucleocapsid (N). These proteins are constituents of the coronavirus virion and have accessory functions in the life cycle of the virus [8,9]. The SARS-CoV-2 genome also encodes the accessory proteins 3ab, 6, 7ab, 8, 9bc, and 10, whose predicted functions are mostly based on sequence similarity with SARS-CoV homologs [9,10]. Consequently, several aspects of the SARS-CoV-2 genome, proteome, and clinical manifestations are still under investigation [11].

The information gained from studying previous coronaviruses has served as a basis for SARS-CoV-2 research [12]. Similar to previous coronaviruses, SARS-CoV-2 mainly causes respiratory illness outbreaks [7], and as such, patients infected with SARS-CoV-2 show symptoms of viral pneumonia, including coughing, breathing difficulties, fever and, in the most severe cases, bilateral lung infiltration. However, a wide range of clinical manifestations has been observed, and in some cases, patients exhibit neurological symptoms [13,14].

Due to the severity of SARS-CoV-2 infection, it is imperative that molecular detection assays be developed. These assays must be adaptable, specific, and sensitive, since this virus is spreading rapidly, continuously receiving evolutionary pressures from the host’s immune system, antiviral drugs, vaccines, and transmission/replication within human populations of different genetic and environmental backgrounds [15,16,17]. SARS-CoV-2 has acquired dozens of mutations and has been classified into a myriad of lineages [18,19,20], highlighting the need to increase global efforts and improve detection strategies to account for the variability of the virus. However, current diagnostic methods can be limited in terms of sensitivity and specificity due to the polymorphic nature of SARS-CoV-2, and thus, there is room for improvement [21,22,23,24].

Therefore, in light of the rapid spread and continuous evolution of SARS-CoV-2, we developed a real-time reverse transcription polymerase chain reaction (real-time RT-PCR) assay utilizing mismatch-tolerant molecular beacons, which can detect this novel coronavirus [25]. The assay incorporates four beacons targeting the S, E, M, and N genes, in addition to an internal positive control (IPC) and synthetic RNA transcripts of the aforementioned gene targets that were used as positive controls. This assay was validated using external quality assessments (EQAs) from the World Health Organization (WHO) and Quality Control for Molecular Diagnostics (QCMD) and clinical samples on the premise of SARS-CoV-2 diagnostic surveillance performed at the University of Cyprus.

## 2. Materials and Methods

### 2.1. Molecular Beacon and Primer Design

**Figure 1:**
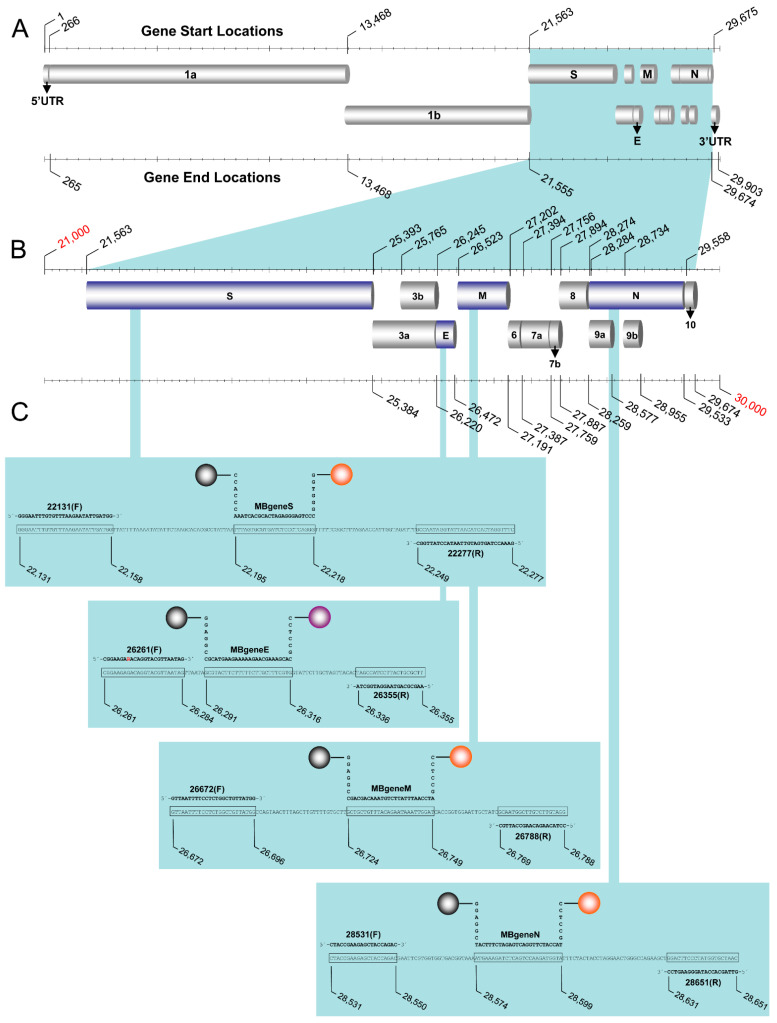
Assay design. Graphic illustration of the genome of SARS-related coronavirus 2 (SARS-CoV-2). (**A**) The genes of the structural proteins targeted in the assay are indicated in the highlighted area. (**B**) The start and end locations of these genes as well as the open reading frames are demonstrated by the nucleotide positions in the magnified area [31]. (**C**) The target genes are further magnified to show the amplicons of each of the four genes (S, E, M, and N) integrated in the assay. The sequences of the primers and molecular beacons are shown in bold, and the target sequences of amplicons are shown in boxes. Numbers that represent the probe, forward, and reverse primers based on the SARS-CoV-2 reference genome (GenBank: MN908947.3) denote the nucleotide positions of the start and end of the target amplicons. The gray spheres (left side of each beacon) indicate the quencher N-[4-(4-dimethylamino)phenylazo]benzoic acid (DABCYL) at the 3′ end of the molecular beacon, while the orange spheres (right side of each beacon) indicate the fluorophore N-HEX-6-aminohexanol (HEX) for the S, N, and M beacons at the 5′ end. The purple sphere indicates the fluorophore N-TET-6-aminohexanol (TET) for the E gene.

**Figure 2:**
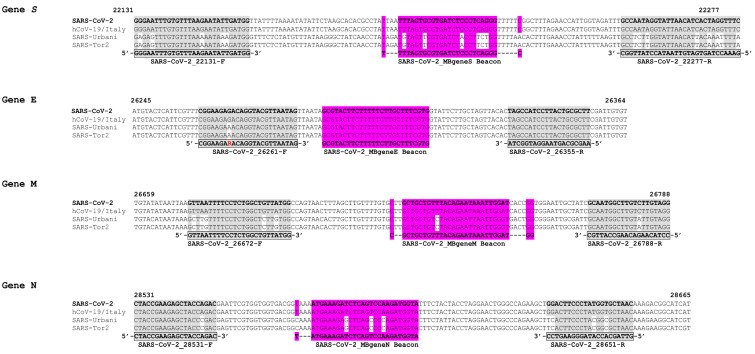
Sequence alignments of the target genes S, E, M, and N. Sequences of gene regions that were used as target areas in the real-time RT-PCR assay to compare SARS-CoV-2-specific genes from SARS-CoV-2 and hCoV-19/Italy strains to those of the pathogenic SARS-CoV strains SARS-Urbani and SARS-Tor2. The corresponding GenBank accession numbers are MN908947.3, MT066156, AY278741 and AY274119, respectively. Sequences in the S, M and N genes were targeted to distinguish SARS-CoV-2 from the rest of the viruses, while the E gene sequences were selected to identify SARS-CoV and SARS-CoV-2 strains. Numbers designate the position of nucleotides with respect to the SARS-CoV-2 genome (GenBank accession no. MN908947.3). The highlighted areas in the alignments show target sequences for the primers and molecular beacons, while areas that are not highlighted within targeted regions signify nucleotides that are not the same as those in the SARS-CoV-2 reference genome (GenBank accession no. MN908947.3).

The molecular beacons, primers, and target amplicons used for this assay were created de novo (Table 1). The assay was designed to detect small regions (95–147 nucleotides) of the SARS-CoV-2 (GenBank accession number MN908947.3) S, N, M, and E genes (Figure 1). Each oligonucleotide was designed using multiple sequence alignments from SARS-CoV and SARS-CoV-2 sequences acquired through the GenBank database (http://www.ncbi.nlm.nih.gov/GenBank, accessed on 26 February 2020): specifically, the pathogenic strains SARS-Urbani (AY278741), SARS-Tor2 (AY274119), and SARS-CoV-2 and the Italian SARS-CoV-2 isolate MT066156 (Figure 2). Sequence alignments were performed using ClustalW [26] and Geneious^®^ 11.1.4 [27] and were used to identify genomic regions in the previously mentioned coronaviruses that were suitable for designing all oligonucleotides pertinent to this assay. The molecular beacons and primers were aimed to detect SARS-CoV-2 regardless of lineage.

The molecular beacons and primers targeting the E gene were designed to identify all SARS-CoV and SARS-CoV-2 sequences used in the alignment, while the S, N, and M genes were used for discriminatory testing specific for SARS-CoV-2. A synthetic IPC was also designed using oligonucleotide sections of the aforementioned genes. The folding and structure of each molecular beacon along with their thermodynamic details were examined using the Mfold deoxyribonucleic acid (DNA) folding webtool [28]. Each molecular beacon was designed so that the stems were six nucleotides long, were complimentary to each other and had a high GC content. Furthermore, the molecular beacons of S, N, M, and E were labeled with the quencher N-[4-(4-dimethylamino)phenylazo]benzoic acid (DABCYL) at the 3′ end and N-HEX-6-aminohexanol (HEX) fluorophore at the 5′ end, except for the E gene, which was labeled with N-TET-6-aminohexanol (TET), and the IPC, which was labeled with 6-carboxy fluorescein (FAM). Imperative to the purposes of this assay was that the loop section of each molecular beacon, which acts as a probe and is complementary to the targeted gene fragment, was designed to be 24–26 nucleotides long, which constitutes the beacons as mismatch tolerant. Whereas, in contrast to allele discrimination, these longer loops are specific and form stable hybrids with their targets, yet their length allows them to be tolerant toward mismatches from mutations that may occur in the genome of SARS-CoV-2 due to recombination and genetic drift [29,30]. The molecular beacons were designed with a high GC content to prevent them from being in an open conformation during PCR in the absence of target. Analysis of the thermodynamic compatibilities of the primers and molecular beacons along with primer self-dimer and heterodimer formation (primer-primer and molecular beacon-primer) were analyzed using the Oligo Analyser tool (IDT, Integrated DNA Technology, Coralville, IA, USA). Through this analysis, the primer-primer and molecular beacon-primer thermodynamic compatibility was evaluated to ensure no cross hybridization between primers and beacons of different genes, since the oligonucleotides (molecular beacons and primers) were designed to have similar melting temperature profiles. It is important to note that in contrast to the molecular beacons, the primers were not designed to be mismatch tolerant, but they were specifically designed to detect SARS-CoV-2. The molecular beacons were synthesized by Biosearch Technologies (Risskov, Denmark), and the primers and other oligonucleotides were synthesized by Macrogen Europe (Amsterdam, The Netherlands) and Biosearch Technologies (Risskov, Denmark).

### 2.2. Internal Positive Control

An internal positive control was developed, which was a synthetic oligonucleotide designed as a mosaic of fragments of the S, E, M, and N genes (Table 1). The IPC amplicon was designed so that it could be amplified by the forward primer for the S gene and the reverse primer for the E gene, while the molecular beacon target sequence was comprised of fragments of the M and N genes. The IPC was compared against the National Center for Biotechnology Information (NCBI) database [32] to ensure that the design was unique and did not recognize other sections of the SARS-CoV-2 genome or the human genome. The IPC was used as a reaction control and it was added during the real time RT-PCR stage.

### 2.3. Thermal Profiles of Molecular Beacons

Melting curve analysis was performed on a 7900HT Fast Real-Time PCR System (Applied Biosystems, Foster City, CA, USA) to assess the thermodynamic characteristics of the molecular beacons. To establish the optimal fluorescence, a variety of molecular beacon concentrations were tested. Ultimately, each reaction volume was 25 μL and consisted of 1.5 μL of MgCl_2_ (50 mM), 2 μL of molecular beacon, 1 μL of a single-stranded oligonucleotide complementary to the target sequence (100 pmol/μL), and 20.5 μL of dH_2_O. To note, the different concentrations that were tested for the molecular beacons were 5, 7.5, and 10 pmol/μL, for a total of 10, 15, and 20 pmol per reaction. The PCR cycling conditions consisted of 1 cycle for 2 min at 95 °C, followed by 50 cycles split in two steps. The first step, during which data were collected, was at 80 °C for 30 s, decreasing by 1 °C per cycle, and the second step was at 80 °C for 10 s, decreasing by 1 °C per cycle. Fluorescence was measured at 535 nm for HEX, 521 nm for TET, and 495 nm for FAM, and the fluorescence was recorded at each cycle. Following the completion of the run, the fluorescence signal data were normalized and plotted.

### 2.4. RNA Transcripts

Synthetic RNA transcripts of each gene target amplicon were developed. These RNA transcripts were transcribed from DNA templates by in vitro transcription using a MEGAscript T7 transcription kit (Ambion, Houston, TX, USA) in accordance with the manufacturer’s specifications. Specifically, to make the RNA transcripts of each gene target, DNA templates were amplified using PCR and forward primers (Table 1) that encompassed the T7 RNA polymerase promoter recognition site (TAATACGACTCACTATAGG) at the 5′ end. For the S, E, M, and N genes, DNA templates were generated using SARS-CoV-2 RNA (BetaCoV/Germany/BavPat1/2020 p.1” grown in cell culture (Charité, Berlin, European Virus Archive goes Global, EVAg)); thus, RT-PCR was performed to generate these templates. However, for the IPC, a single-stranded DNA template was used for the generation of the T7-IPC DNA template.

The volume of each PCR to generate DNA templates was 50 μL, which contained 25.0 μL of 2× Platinum SuperFi RT-PCR Master Mix from the SuperScript IV One-Step RT-PCR System (Thermo Fisher Scientific, Waltham, MA, USA), 12.5 μL of nuclease-free water, 1 μL of 20 pmol/μL each primer, 0.5 μL of SuperScript IV RT Mix (Thermo Fisher Scientific, Waltham, MA, USA) and 10 μL of 10^3^ copies per μL SARS-CoV-2 RNA. Prior to amplification, the RNA was incubated at 70 °C for 20 s and then placed on ice for 1 min. The conditions for the amplification were as follows: 1 cycle for 10 min at 50 °C; 1 cycle for 2 min at 98 °C; 40 cycles of 98 °C for 10 s, 53–56 °C (S: 55 °C, E: 56 °C, M: 53 °C, N: 54 °C) for 10 s and 72 °C for 30 s; and 1 cycle of 72 °C for 5 min. For the IPC RNA transcript, each reaction contained 25 μL of Platinum™ Hot Start PCR 2X Master Mix (Thermo Fisher Scientific, Waltham, MA, USA), 18 μL of nuclease-free water, 1 μL of 20 pmol/μL each primer, and 5 μL of single-stranded IPC DNA template at 10^4^ copies per μL, for a total reaction volume of 50 μL. The conditions of amplification were as follows: 1 cycle at 94 °C for 2 min; 40 cycles at 94 °C for 30 s, 56 °C for 30 s and 72 °C for 30 s; and 1 cycle at 72 °C for 5 min.

The RNA transcripts were purified using NucAway™ Spin Columns Mix (Thermo Fisher Scientific, Waltham, MA, USA). The eluates were then quantified using a NanoDrop^®^ ND-1000 (NanoDrop Technologies, Wilmington, DE, USA). To ensure that there were no traces of DNA template in the RNA transcripts, real-time PCR was performed. Then, real-time RT-PCR was performed to check that the RNA transcripts were properly amplified (as indicated below).

### 2.5. Real-Time RT-PCR: Uniplex and Duplex Reactions

The assay was designed to be used in uniplex and in duplex when each target is run with the IPC. The samples were analyzed by real-time RT-PCR performed on a 7900HT Fast Real-Time PCR System (Applied Biosystems, Foster City, CA, USA) using TaqPath™ 1-Step Multiplex Master Mix (No ROX) (Life Technologies, Frederick, MD, USA). Each 30 μL reaction consisted of 10.0 μL of RNA, 7.5 μL of 4X TaqPath™ 1-Step Multiplex Master Mix, 1.5 μL of 20 pmol/μL each primer, 3.0 μL of 5 pmol/μL molecular beacon, and 6.5 μL of dH_2_O. The primers and molecular beacons for all genes are shown in Table 1. The reverse transcription cycling conditions consisted of 1 cycle at 25 °C for 2 min, followed by 1 cycle at 53 °C for 10 min and 1 cycle at 95 °C for 2 min. This was followed by 5 cycles at 95 °C for 3 s and 53 °C for 30 s. Finally, there were 35 cycles at 95 °C for 3 s and 53 °C for 30 s, when data collection took place. In these runs, no-template controls (NTCs) (dH_2_O) were also added as negative controls, while synthetic RNA transcripts (10^3^ and 10^6^ copies per μL) and SARS-CoV-2 RNA (10^3^ copies per μL) (BetaCoV/Germany/BavPat1/2020 p.1” grown in cell culture (Charité, Berlin, EVAg)) were used as positive controls. The controls were assayed in duplicate for each reaction. Following the completion of the run, the fluorescence signal data were normalized and plotted.

For duplex real-time RT-PCR, the protocol was adapted to account for the increased number of primers and molecular beacons. This protocol was specifically performed for the S, M, and N genes, and each of the genes was amplified with the IPC in the same reaction. The final volume of the reaction was increased to 40 μL, and each contained the following: 10 μL of SARS-CoV-2 RNA at 10^6^ copies/μL (BetaCoV/Germany/BavPat1/2020 p.1” grown in cell culture (Charité, Berlin, EVAg)) or RNA transcripts, 10 μL of IPC target at 10^6^ copies/μL, 7.5 μL of 4× TaqPath™ 1-Step Multiplex Master Mix, 3 μL of the two molecular beacons (5 pmol/μL) in each reaction, 1.5 μL of each primer at 20 pmol/μL, and 0.5 μL of dH_2_O. Notably, for the S-IPC duplex reaction, a new forward primer was designed for the S-gene target, (F) 22,157 (GGTTATTTTAAAATATATTCTAAGCACACGC, 22,157–22,187). For validation of the duplex reactions, a NTC (dH_2_O) was added as a negative control. The targets were also run in uniplex reactions to compare the fluorescence signal between the uniplex and duplex. The cycling conditions remained the same as those in the uniplex real-time RT-PCR. The fluorophores FAM for IPC and HEX for S, M, and N were selected specifically because their excitation wavelengths have little-to-no overlap in their emission spectra. Following the completion of the run, the fluorescence signal data were normalized and plotted.

### 2.6. Validation and Clinical Samples

Validation was accomplished by testing the assay on two external quality assessment (EQA) programs. The first EQA program for the detection of SARS-CoV-2 was hosted by the WHO (WHO SARS-CoV-2 EQAP (2020)). This program provided a panel of five samples of dried, extracted coronavirus RNA (including SARS-CoV-2 RNA in different dilutions and RNA from other currently circulating human coronavirus RNA) as well as a positive control SARS-CoV-2 RNA sample. The second program was from QCMD (QCMD 2020 Coronavirus Outbreak Preparedness EQA Pilot Study), which provided a total of eight samples in transport medium. These programs were designed to evaluate a laboratory’s ability to detect SARS-CoV-2 (COVID-19) using routine molecular methods, and the results were reported during June 2020. 

The assay was further validated under the premise of diagnostic surveillance testing that was performed for students and personnel of the University of Cyprus from September 2020–January 2021 as part of the university’s SARS-CoV-2 surveillance. Nasopharyngeal samples were collected using nasopharyngeal swabs (Biocomma, Shenzhen, China), were placed in tubes with preservation medium (Biocomma, Shenzhen, China), and stored in the fridge (4 °C) for a maximum of 4 h before RNA was extracted. RNA extraction was performed using a QIAmp Viral RNA Mini Kit (Qiagen, Hilden, Germany) and a QIAcube Connect machine (Qiagen, Hilden, Germany) in accordance with the manufacturer’s specifications. Notably, samples were collected using the pooling method, with an average of five samples per pool, and study subjects from positive pools were tested again using new individual samples. Bioethical approval was received by the Cyprus National Bioethics Committee (EEBK EΠ 2020.01.192), and to ensure patient anonymity, all samples were double coded; thus, connections could not be made between the samples and the corresponding study subjects. The collection, processing, and use of samples was in compliance with the rules and regulations of the Cyprus National Bioethics Committee.

## 3. Results

### 3.1. Molecular Beacon Thermal Denaturation Profiles

**Figure 3:**
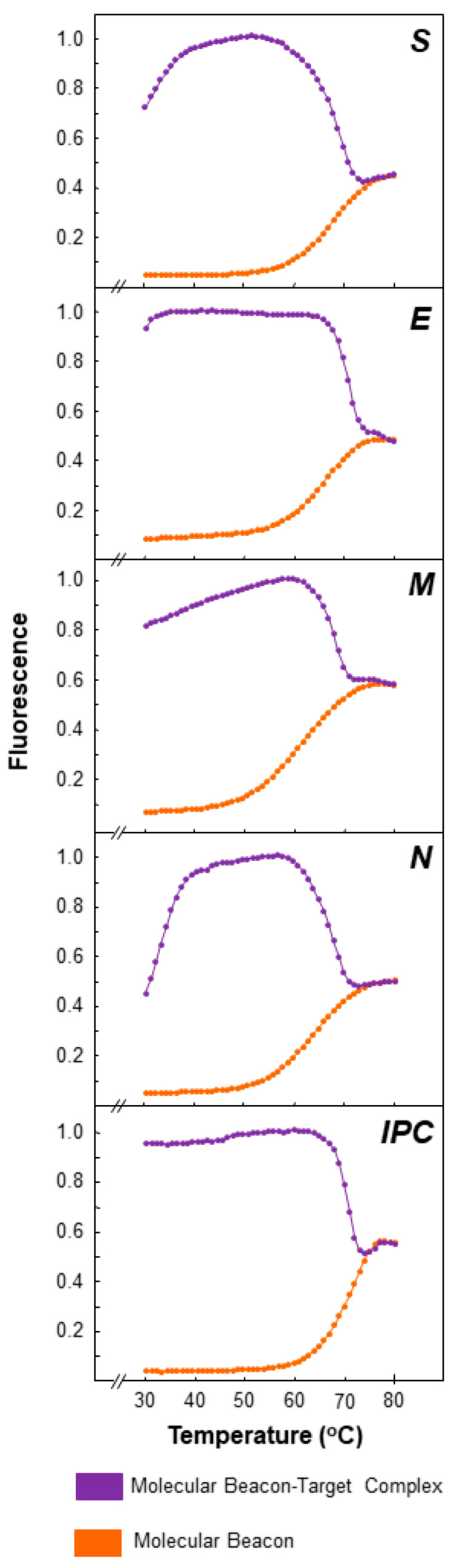
Thermal denaturation profiles of the molecular beacons for S, E, M, and N and the artificial internal positive control (IPC). Melting curve analysis was used to determine the thermal denaturation profiles of the molecular beacons (described in the Materials and methods). The figure demonstrates normalized fluorescence thermal transitions of molecular beacons, found at the bottom part of the plot shown in orange, and beacon-target complexes, found at the top part of the plot shown in purple. The *y*-axis represents the normalized fluorescence data, while the *x*-axis represents the temperature.

For each molecular beacon, the fluorescence for the beacon-target hybrids and for the beacon without target was plotted against temperature to ascertain their thermal characteristics, as well as to identify the appropriate melting temperature for the molecular beacons to hybridize with their targets (Figure 3). As shown in Figure 3, in the absence of the target, the molecular beacons alone displayed a melting temperature of approximately 60 °C, while the largest difference in fluorescence signal between molecular beacons alone and beacon-target hybrids occurred from 35–55 °C. Taking into account the higher fluorescence signal of beacon-target hybrids vs that of beacon alone and the melting temperatures of the primers used in this assay (Table 1), a temperature of 53 °C was selected for the real-time RT-PCRs. Additionally, the optimal concentration that was established for the molecular beacons was 15 pmol per reaction.

### 3.2. Real-Time RT-PCR Validation and Testing

**Figure 4:**
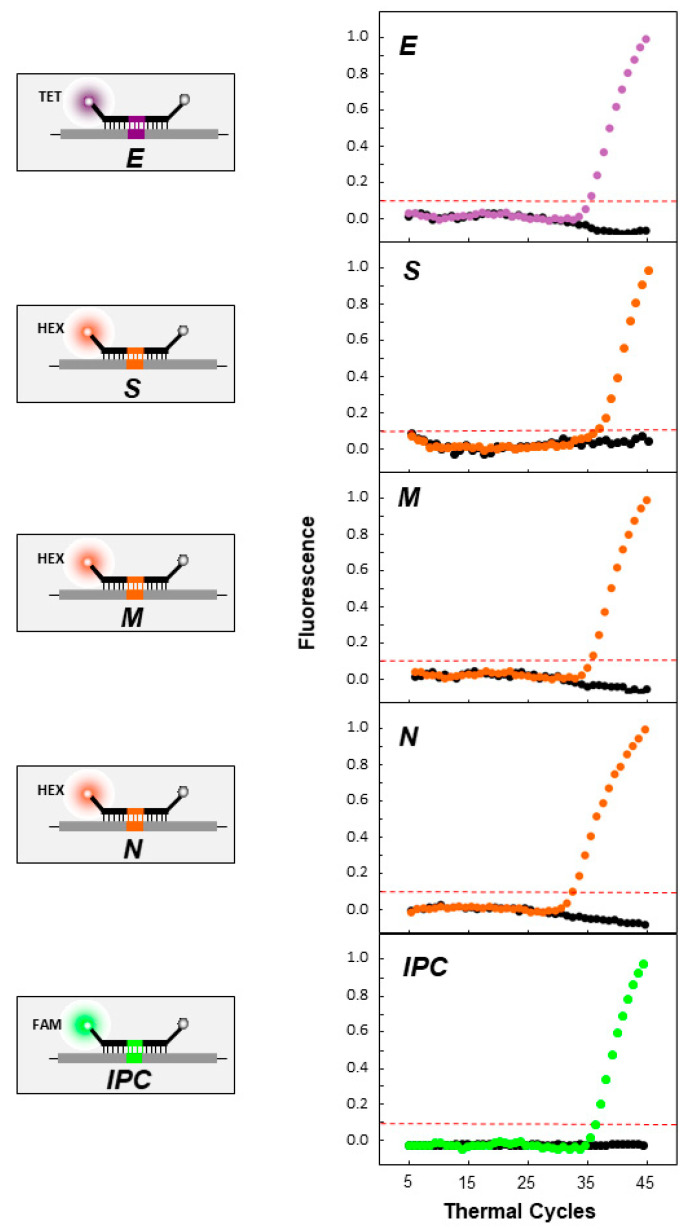
Uniplex real-time RT-PCR of SARS-CoV-2 genes (S, E, M, and N genes) and the artificial IPC. On the left side of the figure, a representation of the quencher and fluorophores carried by each molecular beacon is shown. The gray spheres indicate the quencher DABCYL at the 3′ end of the molecular beacon, while the orange spheres indicate the fluorophore HEX for the S, N, and M beacons at the 5′ end. The purple sphere indicates the fluorophore TET for the E gene. The green sphere indicates the fluorophore 6-carboxy fluorescein (6-FAM) for the IPC. On the right side of each molecular beacon schematic, the fluorescence signal graph is shown for the molecular beacons and their respective targets. The negative control is indicated with black dots. The y-axis represents fluorescence, while the x-axis represents the thermal cycles. The intermittent red line represents the threshold cycle.

**Figure 5:**
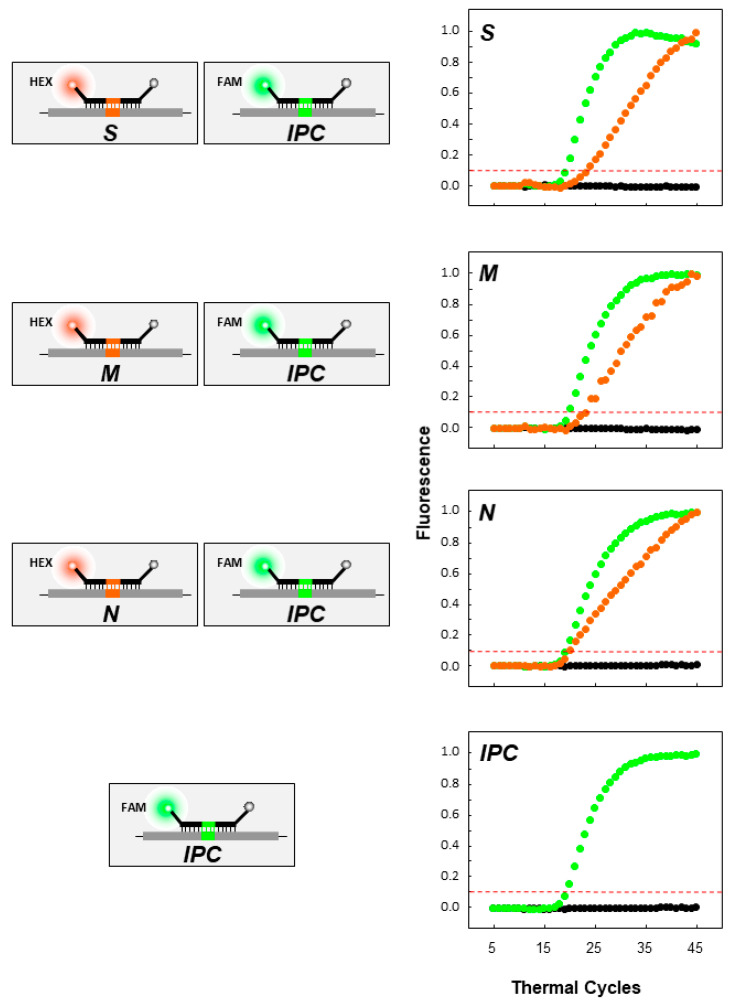
Duplex real-time RT-PCR of SARS-CoV-2 genes (S, M, and N genes) and the artificial IPC. On the left side, a schematic representation of the quencher and fluorophores carried by each molecular beacon (S, M, and N) with the IPC is shown. The gray spheres indicate the quencher DABCYL at the 3′ end of the molecular beacon, while the orange spheres indicate the fluorophore HEX for the S, N and M beacons at the 5′ end. The green sphere indicates the fluorophore 6-FAM for the IPC. On the right side of each schematic of the molecular beacons (S, M, and N) with the IPC, the fluorescence signal graph is shown for the molecular beacons and their respective targets. The negative control is indicated with black dots. The *y*-axis represents fluorescence, while the *x*-axis represents the thermal cycles. The intermittent red line represents the threshold cycle.

The assay was first tested using SARS-CoV-2 RNA (BetaCoV/Germany/BavPat1/2020 p.1” grown in cell culture (Charité, Berlin, Germany, EVAg)) (Figure 4, Table 2). As shown in Figure 4, all target regions were successfully amplified, and the molecular beacons were able to bind to their targets and properly fluoresce, while in the absence of the target (black dots), there was no amplification or fluorescence signal, as expected. Furthermore, for all genes and the IPC, the signal exceeded the threshold at approximately the 32nd–35th cycle. In Figure 5, the duplex reactions of each of the S, M, and N genes in combination with the IPC can be observed. All targets were successfully amplified, and the molecular beacons fluoresced, indicating that there was no cross hybridization and that the duplex reactions did not hinder the results. In the absence of the target, there was no amplification and no fluorescence signal (black dots). The IPC exceeded the threshold at the 19th–20th cycle, while the S, M, and N beacons exceeded the threshold at the 20th–23rd cycle.

Following testing on purified SARS-CoV-2 RNA, two EQAs were used for the validation of the assay, one from the WHO (WHO SARS-CoV-2 EQAP (2020)) and one from QCMD (QCMD 2020 Coronavirus Outbreak Preparedness EQA Pilot Study) (Table 2). For the WHO EQA, the assay successfully identified all blinded samples as well as the positive control. This assay displayed the sensitivity needed to identify the SARS-CoV-2 RNA samples included in the WHO EQA at low, medium, and high concentrations (7.11 × 10^4^, 7.42 × 10^5^ and 1.14 × 10^6^ copies/mL), while it indicated sufficient specificity since it did not identify the negative control and the human coronavirus (hCoV) OC43 RNA. For the QCMD EQA, the assay successfully identified all blinded samples, with SARS-CoV-2 concentrations ranging as low as ~2 × 10^2^ copies/mL and as high as ~2 × 10^5^ copies/mL. Similar to the WHO panel, the assay showed no amplification signal and did not identify as positive hCoV NL63, hCoV OC43 or the negative control in the QCMD EQA panel. Based on the results, the 15 samples included in Table 2, the purified SARS-CoV-2 RNA, and the samples of the two EQA panels, the assay is sensitive enough to detect SARS-CoV-2 in samples containing varying concentrations and specific enough to discriminate SARS-CoV-2 from other human coronaviruses, displaying 100% detection ability and 100% specificity.

For the SARS-CoV-2 surveillance testing performed for students and personnel at the University of Cyprus from September 2020–January 2021, 534 pooled samples were collected, with an average of 5 samples per pool, for a total of 2231 study subjects tested using this assay. From this testing we had identified 22 positive pools and the study subjects from pools that were identified as positive were called in for resampling, and 15 were identified as positive through individual testing using this assay, and an additional one was identified as positive through rapid testing. Importantly, not all targets were required to be performed at once, samples were routinely tested by one target and SARS-CoV-2 positivity was confirmed by a second target, provided the negative and positive controls were correct. The positive controls were in concentrations of 10^3^ copies per μL and 10^6^ copies per μL, were utilized in every run, and the average threshold cycle was 31.7 ± 1.7, and 21.6 ± 1.1 respectively.

## 4. Discussion

The past SARS-CoV epidemic left the future possibility of re-emergence or emergence of other coronaviruses as highly probable [25]. This fact stimulated vigilance toward these viruses and provided us with the basis to develop diagnostic tests faster for other viruses, such as the current SARS-CoV-2 pandemic. The molecular beacon-based real-time RT-PCR assay presented here was originally developed for SARS-CoV by our laboratory [25] (US Patent No. 7,709,188 B2, issued 4 May 2010) and was designed with the capability for adaptation for such viruses and polymorphisms that may arise during the course of their epidemics/pandemics. This rapid and accurate assay, which was modified and further developed for SARS-CoV-2 detection, is an essential tool for the assessment and optimization of disease prevention measures established to safeguard the population, and it can be modified for other infectious agents that harbor a capacity for mutations and constitute a threat to public health.

The sequence identity between SARS-CoV and SARS-CoV-2 has been reported to be 79.5%, and despite this high degree of similarity, there are distinct differences between their genomes, some of which relate to their transmissibility and clinical outcomes [33]. The bioinformatic analysis performed for this study, specifically the multiple sequence alignment, elucidated regions of higher and lower similarity within the structural genes S, E, M, and N between SARS-CoV (Urbani and Tor2) and SARS-CoV-2 (MN908947.3 and MT066156) used for this study (Figure 2). Thus, the region in the E gene that was selected as a target serves as a screening part of the assay because it is conserved among these SARS viruses, and even within SARS-CoV-2, 98.8% of the E protein of SARS-CoV-2 strains worldwide is highly conserved [34]. Similarly, the M protein has also been reported to have a slower rate of evolution and is also highly conserved [35,36]; however, along with the S and N proteins, which tend to evolve faster [36], regions with higher variability were selected for this assay to discriminate between SARS viruses (Figure 2). It is important to note that numerous mutations have already been reported. Taking the S gene as an example, there are at least 8 mutations/deletions in all variants of concern [37], which serve as evidence of the current existing variability that can very well impact current testing methods and show the potential for the accumulation of additional mutations in the near future.

It is therefore important for testing methods to account for the variability in SARS-CoV-2, which arises despite its nsp 14 proofreading capabilities [38]. Nonetheless, SARS-CoV-2 receives evolutionary pressures, and as such, numerous lineages are generated that encompass different sets of mutations and deletions [39]. Reports have already shown that these mutations and deletions compromise SARS-CoV-2 detection assays [21,22,23,24], thereby highlighting the urgent need for the development of screening assays specifically designed to accommodate the polymorphic nature of the virus. A strength of the assay presented in this study has addressed this issue through mismatch-tolerant (“sloppy”) molecular beacon technology [29], which allows for the detection of polymorphisms that may occur in the target sequence. Importantly, the specificity of the beacons is not compromised by a small number mismatched base pairs since the beacon-target duplex is long enough to account for these unexpected polymorphisms [30]. Furthermore, the sensitivity and specificity of the assay is increased since four target regions were selected on four different genes, thereby maximizing the probability of detecting SARS-CoV-2 and minimizing the likelihood of false positive and false negative results. The latter is further supported by both the IPC designed for this assay and the RNA transcript positive controls of each gene.

The present assay exhibited 100% specificity and sensitivity when tested with EQA samples from the WHO and QCMD. It was able to distinguish and identify SARS-CoV-2 from other human coronaviruses, even at borderline concentrations as low as ~2 × 10^2^ copies/mL. However, to improve upon the limitations of this assay, and in order for it to move to clinical or commercial settings, it would require it to be compared with other already available methods [40], as well as a larger cohort to enable more detailed measurements of the limit of detection and precision of the assay. Furthermore, it is important to keep in mind that the pooling method that was utilized for the validation of the assay with the public diagnostic surveillance at the University of Cyprus would be applicable for low prevalence settings. 

Nonetheless, during the testing of this assay at the University of Cyprus for public diagnostic surveillance testing, 16 individual samples were identified as corresponding to SARS-CoV-2 infection out of the 534 pooled nasopharyngeal samples, which were later sequenced as part of a publication regarding a molecular epidemiological study of SARS-CoV-2 infection in Cyprus [41]. In this separate publication, the majority of these 16 samples were classified within the B.1.258 lineage, while the lineages B.1.177 and B.1.1.29 were also identified [41]. Yet, even though certain mutations/deletions, as mentioned above, have been reported to compromise SARS-CoV-2 real-time PCR testing methods, such as ΔH69/V70 [21], which was also found in the B.1.258 lineage, the present assay was unaffected since it did not target those mutated regions of the genome. Nevertheless, the assay was limited by the SARS-CoV-2 lineages that were circulating [41] during the period of the public diagnostic surveillance, and as such, the assay was not applied to a more diverse set of lineages. Moreover, as the pandemic progresses and SARS-CoV-2 is evolving, the assay must be tested with samples from new variants, such as the Delta variant of concern, which was not available during the development and validation of the assay (EQAs June 2020 and public diagnostic surveillance samples September 2020–January 2021). Hence, vigilance must be exercised, and we are regularly checking publicly available genomes from the GISAID database (https://www.gisaid.org/, accessed on 4 October 2021) [42], to ascertain that the number of accumulated mutations does not impact the primers (which are not mismatch tolerant), and is not exceeding the tolerance capabilities of the molecular beacons, ensuring that they can still bind to their targeted regions and consequently detect SARS-CoV-2. Nonetheless, another benefit of the design of this assay is that it provides the advantage of being easily and rapidly adaptable if the above scenario occurs. 

Overall, we present an accurate and reliable uniplex real-time RT-PCR assay, which can also incorporate an IPC. This assay is based on molecular beacons specifically designed to allow for mutations that may arise from the evolutionary pressures that SARS-CoV-2 experiences. This study lays down the foundational work and presents the methodology relevant for this assay to be extrapolated for commercialization and clinical testing at a later stage. This assay offers a fast and efficient way to diagnose SARS-CoV-2 and can serve as a valuable and easily adaptable detection method for current and future mutating infectious agents. 

## Figures and Tables

**Table 1:** Oligonucleotide PCR primers, target amplicons, and molecular beacons used in the real-time PCR assay.

Designation ^1^	TargetGene	Sequence	Position ^2^	Amplicon Length (nts) ^3^	Gene Accession Number ^4^
**PCR Primers**					
22131 (F)	*S*	GGGAATTTGTGTTTAAGAATATTGATGG	22,131–22,158		MN908947.3
22277 (R)	*S*	GAAACCTAGTGATGTTAATACCTATTGGC	22,249–22,277		MN908947.3
26261 (F)	*E*	CGGAAGARACAGGTACGTTAATAG	26,261–26,284		MN908947.3
26355 (R)	*E*	AAGCGCAGTAAGGATGGCTA	26,336–26,355		MN908947.3
26672 (F)	*M*	GTTAATTTTCCTCTGGCTGTTATGG	26,672–26,696		MN908947.3
26788 (R)	*M*	CCTACAAGACAAGCCATTGC	26,769–26,788		MN908947.3
28531 (F)	*N*	CTACCGAAGAGCTACCAGAC	28,531–28,550		MN908947.3
28651 (R)	*N*	GTTAGCACCATAGGGAAGTCC	28,631–28,651		MN908947.3
**Target Amplicons**					
TgeneS	*S*	GGGAATTTGTGTTTAAGAATATTGATGGTTATTTTAAAATATATTCTAAGCACA CGCCTATTAATTTAGTGCGTGATCTCCCTCAGGGTTTTTCGGCTTTAGAACCAT GGTAGATTTGCCAATAGGTATTAACATCACTAGGTTTC	22,131–22,277	147	MN908947.3
TgeneE	*E*	CGGAAGAGACAGGTACGTTAATAGTTAATAGCGTACTTCTTTTTCTTGCTTTCG TGGTATTCTTGCTAGTTACACTAGCCATCCTTACTGCGCTT	26,261–26,355	95	MN908947.3
TgeneM	*M*	GTTAATTTTCCTCTGGCTGTTATGGCCAGTAACTTTAGCTTGTTTTGTGCTTGC TGCTGTTTACAGAATAAATTGGATCACCGGTGGAATTGCTATCGCAATGGCTTG TCTTGTAGG	26,672–26,788	117	MN908947.3
TgeneN	*N*	CTACCGAAGAGCTACCAGACGAATTCGTGGTGGTGACGGTAAAATGAAAGATCTGGTGGTGACGGTAAAATGAAAGATCTCAGTCCAAGATGGTATTTCTACTACCTA GGAACTGGGCCAGAAGCTGGACTTCCCTATGGTGCTAAC	28,531–28,651	121	MN908947.3
TIPC	N/A	GGGAATTTGTGTTTAAGAATATTGATGGTTAGCTGCTGTTTACAGTCCAAGATG GTAGTATTCTTGCTAGTTACACTAGCCATCCTTACTGCGCTT	N/A	96	N/A
**Molecular Beacons** ^5^					
MBgeneS	*S*	HEX-GGTGGGCCCTGAGGGAGATCACGCACTAAACCCACC-DABCYL	22,195–22,218		MN908947.3
MBgeneE	*E*	TET-CCTCCGCACGAAAGCAAGAAAAAGAAGTACGCCGGAGG-DABCYL	26,291–26,316		MN908947.3
MBgeneM	*M*	HEX-CCTCCGATCCAATTTATTCTGTAAACAGCAGCCGGAGG-DABCYL	26,724–26,749		MN908947.3
MBgeneN	*N*	HEX-CCTCCGTACCATCTTGGACTGAGATCTTTCATCGGAGG-DABCYL	28,574–28,599		MN908947.3
MBIPC	N/A	FAM-GCCCACGTACCATCTTGGACTGTAAACAGCAGCCGTGGGC-DABCYL	N/A		N/A

^1^ PCR primer, oligonucleotide, and molecular beacon names as they appear in the text; the orientation of the PCR primer is indicated in parentheses: F, forward; R, reverse. ^2^ Positions correspond to the appropriate GenBank sequences; in the case of molecular beacons, the positions correspond to the target recognition sequences of the molecular beacon probe. ^3^ Amplicon length denotes the size in nucleotides (nts) of the target amplicons for each of the four genes (S, E, M, N) as well as the internal positive control (IPC). ^4^ The specific SARS-CoV-2 genome that was employed as a reference to design the oligonucleotides used in this assay can be found in GenBank (https://www.ncbi.nlm.nih.gov/genbank/, accessed on 26 February 2020) under accession number MN908947.3. ^5^ Underlined regions denote the stem of the molecular beacons; FAM denotes 6-carboxy fluorescein; TET, N-TET-6-aminohexanol; HEX, N-HEX-6-aminohexanol; and DABCYL, N-[4-(4-dimethylamino)phenylazo]benzoic acid.

**Table 2:** Real-time RT-PCR results of human SARS-CoV-2 specimens.

Sample	ID of Specimen Analyzed	RT-PCR Result ^4^			
		*S*	*E*	*M*	*N*
**EVAg** ^1^					
1	Purified RNA of SARS-CoV-2 cell culture supernatant	+	+	+	+
**WHO** ^2^					
2	2020-01 (SARS-CoV-2)	+	+	+	+
3	2020-02 (SARS-CoV-2)	+	+	+	+
4	2020-03 (NEGATIVE)	−	−	−	−
5	2020-04 (OC43)	−	−	−	−
6	2020-05 (SARS-CoV-2)	+	+	+	+
7	Positive control (SARS-CoV-2)	+	+	+	+
**QCMD** ^3^					
8	CVOP20S2-01 (SARS-CoV-2)	+	+	+	+
9	CVOP20S2-02 (NL63)	−	−	−	−
10	CVOP20S2-03 (SARS-CoV-2)	+	+	+	+
11	CVOP20S2-04 (OC43)	−	−	−	−
12	CVOP20S2-05 (NEGATIVE)	−	−	−	−
13	CVOP20S2-06 (SARS-CoV-2)	+	+	+	+
14	CVOP20S2-07 (SARS-CoV-2)	+	+	+	+
15	CVOP20S2-08 (SARS-CoV-2)	+	+	+	+

^1^ EVAg, European Virus Archive goes Global. ^2^ WHO, World Health Organization, External Quality Assessment Program for the Detection of SARS-CoV-2 by RT-PCR (2020) (WHO SARS-CoV-2 EQAP (2020)). Program code SARS-CoV-2-EQAP-01. ^3^ QCMD, Quality Control for Molecular Diagnostics, QCMD 2020 Coronavirus Outbreak Preparedness EQA Pilot Study. Program code CVOP20. ^4^ Detectable real-time PCR amplification signal is denoted by the symbol (+), which indicates consistent C_T_ values ≤ 45 for each of the four SARS-CoV genes assayed in this experiment, including an internal positive control (IPC). An undetectable signal (C_T_ values > 45) is denoted by (−) and indicates the absence of template cDNA.

## Data Availability

The results will be available upon request from the corresponding author.
